# A phase I followed by a randomized phase II trial of two cycles carboplatin-olaparib followed by olaparib monotherapy versus capecitabine in *BRCA1*- or *BRCA2*-mutated HER2-negative advanced breast cancer as first line treatment (REVIVAL): study protocol for a randomized controlled trial

**DOI:** 10.1186/s13063-016-1423-0

**Published:** 2016-06-21

**Authors:** Philip C. Schouten, Gwen M. H. E. Dackus, Serena Marchetti, Harm van Tinteren, Gabe S. Sonke, Jan H. M. Schellens, Sabine C. Linn

**Affiliations:** Department of Molecular Pathology, Antoni van Leeuwenhoek Hospital - Netherlands Cancer Institute, Plesmanlaan 121, 1066CX Amsterdam, The Netherlands; Department of Pathology, Utrecht University Medical Center, Heidelberglaan 100, 3584CX Utrecht, The Netherlands; Division of Medical Oncology, Antoni van Leeuwenhoek Hospital - Netherlands Cancer Institute, Plesmanlaan 121, 1066CX Amsterdam, The Netherlands; Department of Clinical Pharmacology, Antoni van Leeuwenhoek Hospital - Netherlands Cancer Institute, Plesmanlaan 121, 1066CX Amsterdam, The Netherlands; Department of Biometrics, The Netherlands Cancer Institute, Plesmanlaan 121, 1066CX Amsterdam, The Netherlands; Faculty of Science, Utrecht Institute of Pharmaceutical Sciences (UIPS), Universiteitsweg 99, 3584CG Utrecht, The Netherlands

**Keywords:** Breast cancer, Metastatic, *BRCA1*, *BRCA2*, Homologous recombination deficiency, PARP inhibitors, Olaparib, Carboplatin, Capecitabine

## Abstract

**Background:**

Preclinical studies in breast cancer models showed that *BRCA1* or *BRCA2* deficient cell lines, when compared to *BRCA* proficient cell lines, are extremely sensitive to PARP1 inhibition. When combining the PARP1 inhibitor olaparib with cisplatin in a *BRCA1*-mutated breast cancer mouse model, the combination induced a larger response than either of the two compounds alone. Several clinical studies have investigated single agent therapy or combinations of both drugs, but no randomized clinical evidence exists for the superiority of carboplatin-olaparib versus standard of care therapy in patients with *BRCA1*- or *BRCA2*--mutated metastatic breast cancer.

**Methods/design:**

This investigator-initiated study contains two parts. Part 1 is a traditional 3 + 3 dose escalation study of the carboplatin-olaparib combination followed by olaparib monotherapy. The carboplatin dose will be escalated from area under the curve (AUC) 3 to AUC 4 with an olaparib dose of 25 mg BID. Olaparib is subsequently escalated to 50, 75, and 100 mg BID until >1/6 of patients develop dose-limiting toxicity (DLT). The dose level below will be the maximum tolerable dose (MTD). It is expected that 15–20 patients are needed in Part I.

In Part 2 *BRCA1*- or *BRCA2*-mutated HER2-negative breast cancer patients will be randomized between standard capecitabine 1250 mg/m^2^ BID day 1–14 q day 22, versus 2 cycles carboplatin-olaparib followed by olaparib monotherapy 300 mg BID. In total 104 events in 110 patients need to be observed to detect a 75 % clinically meaningful improvement in progression-free survival (PFS), from a median of 4 months (control) to 7 months (experimental) assuming a 2-year accrual and ≥6 months of follow-up with 80 % power (5 %, two-sided significance level). After progression on first line treatment, patients will receive physician’s best choice of paclitaxel, vinorelbine, eribulin, or capecitabine (experimental arm only) at standard dose. A compassionate use program of olaparib is available for patients in the standard arm after progression on second line treatment.

**Discussion:**

Results might be pivotal for registration of olaparib as standard first line treatment in advanced *BRCA1*- or *BRCA2*-mutated breast cancer.

**Trial registration:**

ClinicalTrials.gov identifier: NCT02418624. Registered on 9 March 2015. EudraCT number: 2013-005590-41. Registered on 15 October 2014. Protocol version 3.0.

**Electronic supplementary material:**

The online version of this article (doi:10.1186/s13063-016-1423-0) contains supplementary material, which is available to authorized users.

## Background

Patients harboring inactivating mutations in *BRCA1* or *BRCA2* are at an increased risk of developing breast cancer [1]. It has become clear that these genes are important in error-free DNA double strand break (DSB) repair via homologous recombination. Loss of the second allele inactivates error-free repair of DNA DSBs. Error-prone mechanisms of repair substitute and repair these DNA lesions, resulting in either aberrations in the genome or cell death when crisis cannot be resolved [2–8]. Targeting the defective repair with DNA double strand break-inducing agents like platinum compounds might be beneficial for *BRCA1* or *BRCA2* mutation carriers with breast cancer. Single agent carboplatin has been extensively investigated, from fundamental studies to clinical studies, e.g., the Triple Negative Breast Cancer trial (TNT, NCT00532727) enriched for patients with cancers harboring *BRCA* mutations. In the TNT trial, patients with advanced triple negative (TN) breast cancer were randomized to six 3-weekly cycles of carboplatin area under the curve (AUC) 6 or six 3-weekly cycles of docetaxel 100 mg/m^2^ in first or second line. The TNT trial showed that patients with a *BRCA1* or *BRCA2* mutation derive benefit from carboplatin over docetaxel [9]. However, carboplatin is not a registered choice for this subgroup at the moment.

Another way to target defective homologous recombination is by inhibition of poly(ADP)ribose polymerase-1 (PARP1) [10–12]. *BRCA1* or *BRCA2* deficient cells and conditional mouse tumors proved to be extremely sensitive to PARP1 inhibition in clonogenic survival assays, whereas *BRCA* proficient cells were not sensitive [10–12]. PARP1 inhibition may result in cell kill through different mechanisms: (1) the inhibition of single strand break repair resulting in single strand breaks evolving into DSBs which cannot be repaired error-free in the absence of BRCA, (2) trapping of PARP1 on damaged DNA, (3) impairment of BRCA1 recruitment, or (4) the activation of non-homologous end joining [13–17]. Olaparib is a PARP1 inhibitor that has recently been approved by the European Medicines Agency (EMA) as maintenance therapy for *BRCA*-mutated advanced high-grade serous ovarian cancers after response to platinum-based chemotherapy. The FDA approved olaparib for the treatment of advanced *BRCA*-mutated ovarian cancers previously treated with ≥3 lines of chemotherapy [18, 19].

Several phase I and II clinical trials have tested olaparib monotherapy as capsule formulation in (breast) cancer populations carrying a *BRCA1* or *BRCA2* mutation or populations enriched for mutation carriers [20–23]. Patients were required to take 16 olaparib capsules of 50 mg each day to reach the 400-mg BID monotherapy dose. Therefore, a tablet formulation was developed. The capsule and tablet formulations are not bio-equivalent. The oral pharmacokinetics of olaparib as capsule formulation was nonlinear. The tablet formulation was tested and found safe. Four hundred mg BID olaparib capsules are dose equivalent to 2 tablets of 150 mg olaparib (300 mg BID) [24–26].

One study enrolled 60 patients in a phase I trial. The maximum tolerable dose (MTD) of olaparib as capsule formulation was found to be 400 mg BID. Maximum PARP1 inhibition was reached at doses of 100 mg. In total 12/19 *BRCA1* or *BRCA2* mutation carriers derived clinical benefit [21]. In a follow-up study, 81 patients were screened for *BRCA1* or *BRCA2* mutations, and 54 of them were enrolled into receiving 100 mg BID or 400 mg BID, respectively. The primary outcome was objective response rate (ORR) according to the Response Evaluation Criteria In Solid Tumors (RECIST). Intra-patient dose escalation was allowed since interim analyses showed that responses in the 400 mg cohort were more durable. Despite maximum PARP1 inhibition at 100 mg BID, the median progression-free survival was 5.7 months in the 400 mg BID cohort versus 3.8 months in the 100 mg BID cohort [22]. Adverse events occurred in up to 81 % of patients and were mostly mild, grade 1 or 2 according to Common Terminology Criteria for Adverse Events (CTCAE) and consisted of nausea, vomiting, fatigue, and myelosuppression [21–23].

As mentioned, both traditional platinum drugs and olaparib target a defect in homologous recombination, platinum agents by directly inducing toxic DNA lesions and olaparib by (a combination of) inhibiting backup repair pathways, PARP1 trapping, impairing BRCA1, or activating non-homologous end joining (NHEJ) with a synthetic lethal result. Combining the two may therefore induce extra benefit for patients. In fact, in *BRCA2* deficient cells it was found that the combination of olaparib and cisplatin was synergistic [27]. Furthermore, the combination of cisplatin with olaparib was investigated in a *BRCA1*-associated breast cancer mouse model. In this model combination treatment induced more durable responses compared to either of the compounds alone [12]. The strategy of combining multiple drugs has potentially a more than additive effect. Although the combination is limited by bone marrow toxicity, it may be that the combination of two types of damage is more effective than overloading a cell with only one type of damage mechanism. Furthermore, sensitization to olaparib may occur, for example, due to DNA damage, inducing PARP1 expression [16, 17, 28].

The non-randomized open-label phase I study NCT00516724 investigated combinations of olaparib with carboplatin and/or paclitaxel in advanced breast and ovarian cancer. A final report has not been published yet, but pharmacokinetic (PK) and toxicity information was available to guide the design of the current trial. Many cohorts in this trial demonstrated dose-limiting toxicities early in the six cycles of combination treatment and required dose modifications. These findings are similar to other trials, in which the selected schedule did not contain continuous dosing of olaparib [29, 30]. Therefore, cohorts without dose-limiting toxicity or treatment delays in the first two cycles were deemed feasible regimens for combination therapy. These feasible dose levels comprised carboplatin AUC 4 in combination with either olaparib 50 mg BID plus paclitaxel 90 mg/m^2^ or olaparib 50 mg BID plus paclitaxel 75 mg/m^2^ or olaparib 200 mg BID on days 1–10 plus paclitaxel 175 mg/m^2^. The aforementioned regimens contained olaparib capsules and all regimens contained paclitaxel. For these two reasons a brief dose-finding study of carboplatin in combination with olaparib tablets is required. In an analysis of anti-tumor activity, cohorts were grouped by olaparib formulation and drug combination. In these cohorts response rates determined as stable disease (SD) >2 months, partial response (PR), or complete response (CR) were 38 % (16/42) among patients [31]. Despite the limitations of a single arm trial testing six cycles of combination therapy, extrapolating this to a new study with modified and probably improved drug formulation and combination treatment (no paclitaxel and fewer combination cycles) seems feasible, since doses are exerting effects both in inducing toxicity and preliminary responses. After completing combination therapy, patients remained on olaparib monotherapy, sometimes for very long periods of time [32]. One may wonder whether combining carboplatin and olaparib only in the first two cycles may be insufficient to yield benefit. However, the response and toxicity observed in the NCT00516724 trial suggest that the proposed dose escalation scheme will not lead to subtherapeutic dosing despite incorporating carboplatin only in the first two cycles. For the monotherapy dose, we follow previous studies that established 300 mg BID as being safe and efficacious [24–26].

Currently no specific guidelines for systemic therapy in the metastatic setting are available for *BRCA1*- or *BRCA2*-mutated breast cancers. Capecitabine is registered for the indication of metastatic breast cancer if patients have been pretreated with anthracyclines and a taxane and does not specifically target a defect in homologous recombination [33, 34].

No randomized comparative evidence supporting olaparib monotherapy or combination therapy in metastatic breast cancer is available, but sufficient evidence is available to conduct such a trial. It is considered of importance to swiftly determine whether a *BRCA1* or *BRCA2* mutation targeting regimen has a positive benefit/risk ratio. Therefore, we initiated this combined phase I/randomized phase II controlled trial.

## Methods/design

**Fig. 1:**
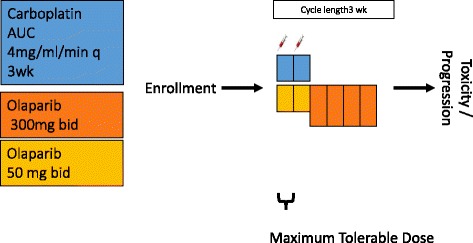
Schematic representation of the study. In Part 1 patients with advanced disease will be enrolled to be administered carboplatin and olaparib in two 3-weekly cycles followed by olaparib monotherapy until dose limiting toxicity or progressive disease. The maximum tolerable dose will be determined

**Fig. 2:**
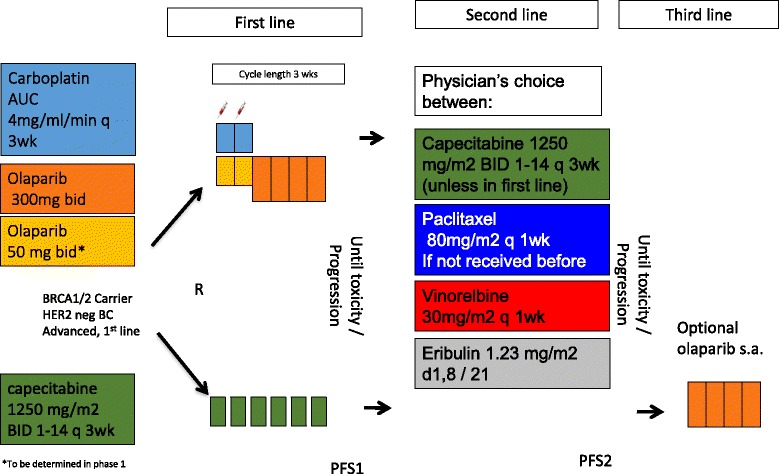
Patients with *BRCA1*- or *BRCA*2-mutated HER2-negative, advanced breast cancer will be randomized to carboplatin-olaparib (based on the Part 1 results) or capecitabine. After progression on first line, patients will be administered physician’s best choice out of capecitabine, paclitaxel, vinorelbine, or eribulin to assess progression-free survival on second line treatment. For patients who were not randomized to olaparib, a rollover protocol is available for third line treatment in the advanced setting

A description of this manuscript according to the SPIRIT guidelines is presented in Additional file 1. This investigator-initiated study consists of two parts. During Part 1, a traditional 3 + 3 dose escalation study is performed to determine the MTD of two cycles carboplatin-olaparib followed by olaparib. This is required due to a change in formulation from olaparib capsules to olaparib tablets with improved pharmacological characteristics. After the MTD is established in Part 1, Part 2 will be initiated. Part 2 is a phase II randomized, multicenter, open-label trial, comparing the progression-free survival on first line treatment (PFS1) of two cycles of carboplatin-olaparib followed by olaparib monotherapy to capecitabine monotherapy. After progression on first line treatment, patients will receive physician’s best choice of paclitaxel, vinorelbine, or eribulin, and in the case of carboplatin-olaparib treated patients, the same choice complemented with capecitabine. A schematic description of the trial is shown in Figs. 1 and 2. A rollover protocol is in place for patients who were not randomized to olaparib in first line to allow them access to olaparib monotherapy as third line treatment. Centers participating in Part 2 of the study are currently being recruited.

### Objectives

The primary objective of Part 1 is to determine the MTD of two cycles carboplatin-olaparib and one cycle olaparib monotherapy at standard dose. The MTD is defined as the dose level below the dose level at which >1/6 of patients experience a dose-limiting toxicity (DLT). The secondary objectives are to investigate the systemic exposure of the olaparib tablet formulation, the pharmacodynamics (PD) of olaparib, and the ORR of carboplatin and olaparib according to RECIST.

In Part 2 the primary objective is to compare the PFS1 of two cycles olaparib-carboplatin followed by olaparib to capecitabine. Secondary objectives are to compare the ORR, the progression-free survival on second line treatment (PFS2), and overall survival with either two cycles of olaparib-carboplatin followed by olaparib or capecitabine as first line and to determine treatment safety in first and second lines. In both Parts 1 and 2 additional tissue and blood will be collected for exploratory biomarker analyses.

### Inclusion criteria

Patients are eligible for this study if they are ≥18 years old, have histological or cytological proven metastatic cancer, with a life expectancy ≥3 months, a World Health Organization (WHO) performance status of 0, 1, or 2, and a negative pregnancy test. Minimal acceptable safety laboratory values include ANC ≥1.5 × 10^9^/L, hemoglobin ≥6.2 mM (with no transfusions in the last 28 days), platelet count ≥100 × 10^9^/L, serum bilirubin ≤1.5 × upper limit of normal (ULN) (or <3 × ULN in case of known Gilbert’s syndrome), ASAT and ALAT 2.5 × ULN (or <5 × ULN in case of liver metastasis), and serum creatinine ≤1.5 × ULN or creatinine clearance ≥50 mL/min (by the Cockcroft-Gault formula). Patients should be able and willing to give informed consent.

In Part 1, all patients with tumors who may benefit from carboplatin-olaparib therapy and who did not receive more than one line of systemic chemotherapy in the advanced setting and any line of hormonal therapy for advanced disease may be included. Prior (neo-)adjuvant chemotherapy is accepted and does not count as one line, because it was administered for early stage disease. The tumor must be evaluable according to RECIST 1.1. Patients must consent to undergo blood sampling for pharmacokinetics and pharmacodynamics analyses.

In Part 2, patients must have *BRCA1*- or *BRCA2*-mutated HER2-negative breast cancer. Pretreatment should consist of an anthracycline and taxane in the (neo-)adjuvant setting (unless not indicated) following the label for capecitabine monotherapy in the metastatic setting but without systemic chemotherapy pretreatment for advanced disease and with maximally two lines of hormonal therapy pretreatment in the advanced setting. The tumor must be measurable according to RECIST 1.1.

### Exclusion criteria

Patients are excluded from this study if they are pregnant or breastfeeding, use unreliable contraceptive methods, are treated with cytochrome P450 3A4 (CYP3A4) inducers or inhibitors, or previously received a PARP1 inhibitor or high-dose alkylating agent. Radiotherapy and treatment with investigational drugs are prohibited within 28 days prior to receiving the first dose of investigational treatment; exceptions are 1 × 8 Gray (Gy) for pain palliation and standard (neo-)adjuvant chemo-, hormonal- and immunotherapy. Then 7- and 21-day intervals should be maintained, respectively. Other conditions excluding a patient from participating in this part of the study are uncontrolled infectious diseases, known human immunodeficiency virus (HIV)-1 or −2 infections, active hepatitis B or C, myocardial infarction (<6 months) or unstable angina, myelodysplastic syndrome, acute myeloid leukemia, symptomatic brain metastases, leptomeningeal metastases, or any other medical condition that interferes with study procedures or compliance and/or jeopardizes safe treatment.

In Part 1 patients are excluded if they previously received carboplatin, unless no progression on carboplatin was observed during earlier treatment and the last carboplatin administration was longer than 6 months ago.

In Part 2 patients are excluded if they are dihydropyrimidine dehydrogenase (DPD) deficient, received treatment for advanced disease with non-hormonal anticancer therapy or >2 lines of endocrine therapy, or were pretreated with capecitabine. Pretreatment should include an anthracycline and taxane in the (neo-)adjuvant setting (unless not indicated) following the label for capecitabine monotherapy in the metastatic setting.

### Interventions

In Part 1, patients will receive two cycles olaparib-carboplatin followed by olaparib monotherapy. Treatment cycles will be 21 days with the exception of cycle 1, which contains a day 0 for olaparib PK measurements. Carboplatin will be administered intravenously on day 1 of cycles 1 and 2. A single oral dose of olaparib is administered on day 0 and 1 of cycle 1; afterwards olaparib administration will be twice daily. After cycle 2 olaparib monotherapy is given at 300 mg BID until progression or unacceptable treatment-related toxicity occurs. Patients will be screened for baseline inclusion criteria, and after informed consent has been received, baseline safety measurements will be obtained. Subsequently, patients will be followed up with hospital visits for safety assessment comprising recording of medical history, and physical examination and laboratory testing every week in cycles 1 and 2, every cycle in cycles 3 and 4, and every two cycles from cycle 5 onwards, unless otherwise indicated. Tumor assessment by RECIST will be performed every two cycles. PK/PD measurements are taken in cycle 1. Extra blood and tissue samples are collected if informed consent is obtained before and after treatment.

Dose escalation is performed according to a traditional 3 + 3 design. The carboplatin dose will be escalated in one step from AUC 3 to AUC 4 with a constant olaparib dose of 25 mg BID. Olaparib is then escalated to 50, 75, and 100 mg BID until >1/6 patients develop a DLT; the previous safe dose level will be determined as the MTD and is used as the treatment schedule in Part 2. In Part 2 two cycles carboplatin-olaparib followed by olaparib monotherapy (experimental arm), dosed according to the study results of Part 1, are compared to registered standard of care capecitabine (1250 mg/m^2^ BID d 1–14 in 21-day cycles, according to the label). After progression, patients will receive physician’s choice of intravenously delivered (IV) eribulin (1.23 mg/m^2^ d1,8/21, equivalent to 1.4 mg/m^2^ of eribulin mesylate), vinorelbine (IV) (25 mg/m^2^ q 1wk), paclitaxel (IV) (80 mg/m^2^ q 1wk), or capecitabine (if randomized to carboplatin-olaparib as first line treatment), drugs registered for second line treatment of metastatic breast cancer. For patients randomized to the standard arm, olaparib will be available as third line treatment at time of disease progression.

Patients will be screened for baseline inclusion criteria and, after signing informed consent, baseline safety measurements will be obtained. Subsequently, patients will be followed with hospital visits for safety assessment comprising medical history taking, physical examination, and laboratory testing every cycle from cycle 1 until 4, and minimally every two cycles from cycle 5 onwards, unless more visits are medically indicated. Tumor assessment by RECIST will be performed every two cycles.

### Dose modifications

Dose modifications are allowed when toxicity CTCAE grade >2 (or intolerable grade 2, despite supportive care) has resolved to grade <2. During carboplatin-olaparib combination therapy the carboplatin dose will not be modified. The olaparib dose may be modified in steps of 25 mg following the inversed dose escalation scheme to a minimum of 25 mg BID. For the first toxicity developing after the second cycle of carboplatin, the olaparib dose does not have to be modified and treatment continues when the toxicity has resolved. However, if a second toxicity develops after the second carboplatin dose, the dose of olaparib must be modified. Patients who develop toxicity while receiving olaparib monotherapy 300 mg BID can resume treatment at 250 mg BID and subsequently 200 mg BID once adequately recovered. Dose modifications for capecitabine [34], eribulin [35], paclitaxel [36], and vinorelbine [37] are allowed according to prespecified steps that follow the label.

### Pharmacokinetics (PK) and pharmacodynamics (PD)

Blood samples for olaparib and/or carboplatin PK will be drawn in Part 1 cycle 1 to capture the single agent, single-dose olaparib PK curve and the PK curves of olaparib and carboplatin in combination treatment. The amount of PARP1 inhibition will be measured in peripheral blood mononuclear cells (PBMCs) before and during olaparib treatment.

### Concomitant treatment

Anti-emetic treatment will be administered before and following the administration of carboplatin, and if necessary, with erubilin, paclitaxel, and vinorelbine. Patients receiving paclitaxel should be pretreated to prevent allergic reactions. Supportive care and other medication may be given at the discretion of the investigator(s) and according to good clinical practice. Administration of granulocyte colony-stimulating factor (GCSF) is allowed, but not during the first treatment cycle. One time 8 Gy palliative radiation at focal sites (preceded and followed by a 3-day wash-out period) and bone targeting agents for the treatment of bone metastases are allowed. CYP3A4 inhibitors or inducers are not allowed in Part 1 and not advised in Part 2. CYP2C8 inhibitors or inducers are not advised in Part 2.

### Safety assessments/participant timeline

Measurements used to evaluate safety will include assessment of signs and symptoms/adverse events, physical examination, performance status, blood pressure and heart rate measurements, clinical laboratory testing (hematology, clinical chemistry, and urinalysis), and 12-lead electrocardiography (ECG) monitoring. Patients are evaluable for safety if they received carboplatin intravenously and a dose of olaparib.

### Statistics: sample size

For Part 1 no sample size calculation has been performed as statistical analyses will be descriptive in nature. From previous trials it is estimated that 15–20 patients are needed to establish the MTD. In Part 2, based on expert opinion, the sample size was calculated using the following parameters: median PFS for the control group of 4 months and considering 3 months a worthwhile improvement (hazard ratio of 1.75 under the alternative hypothesis). Further, choosing a two-sided type I error of 0.05, a total of 104 events need to be observed guaranteeing a power of 80 %. Assuming a constant accrual over 2 years and another 6 months of follow-up after the last patient entered (1:1 randomization), 110 patients need to be enrolled. An interim analysis is included allowing for early stopping. The spending function selected is O’Brien-Fleming for the upper bound (efficacy) and the Hwang-Shih-DeCani spending function with parameter −1 for the non-binding lower bound for futility [38]. Sample size calculations were performed using the gsDesign package in R [39]. Regarding the assumptions on survival, these are based on a mostly triple negative population, due to *BRCA1*-mutated cancers, and a 4–5 month expected benefit of capecitabine depending on the baseline characteristics, with a reported smaller time to progression of triple negative patients [40–44]. Patients will be stratified by treating center and by hormone receptor status. Given the assumptions, it is expected that the interim analysis occurs after approximately 16 months and the final analysis after 30 months. If these assumptions are not met during the trial, the optimal course of adjustment will be decided in collaboration with an independent Data and Safety Monitoring Board. No sample size calculation was performed for second line treatment.

### Statistics: methods

In Part 1 the baseline characteristics of the study population will be described according to common reporting standards (CONSORT 2010 guidelines [45]). Patients are considered evaluable for PK analyses if they underwent PK blood sampling during cycle 1. The pharmacokinetics will be determined using non-compartmental methods. The mean, median, coefficient of variation, and range of the following carboplatin and olaparib parameters will be calculated: time to maximal plasma concentration (t_max_), maximum plasma concentration (C_max_), area under the time versus concentration curve from zero to the last data point (AUC_0-t_), area under the time versus concentration curve from zero to infinity (AUC_inf_), half-life (t_1/2_), mean residence time (MRT), volume of distribution (Vdd), and clearance (Cl). To compare the AUC of capsules obtained from previous studies to the tablet AUC obtained in this study, the ratio of log(AUC-tablet)/log(AUC-capsule) should be between 0.8 and 1.25. Inhibition of PAR will be measured with a PAR activation assay on PBMCs and will be presented as the percentage of activation after olaparib treatment compared to activation before olaparib treatment. A final descriptive analysis for response, based on RECIST 1.1, will be done when the last patient has completed the follow-up visit at end of treatment.

In Part 2 baseline characteristics of the study population will be described according to common reporting standards (CONSORT 2010 guidelines [45]). PFS1 between the experimental and control arms will be assessed on the intention-to-treat population using the Kaplan-Meier survival estimate and log rank test. A Cox proportional hazards model is used to assess treatment effect size and adjust for confounding factors. The data cut-off date will be when 104 events have been observed. Patients will be followed up to collect additional survival and safety data for PFS2, and updated safety and efficacy analyses will be performed.

### Logistics and administrative arrangements

REVIVAL was approved by the accredited Medical Ethics Committee of the Netherlands Cancer Institute - Antoni van Leeuwenhoek Hospital (NKI-AVL) on 10 February 2015. The study protocol follows the principles of the Declaration of Helsinki and the Medical Research Involving Human Subjects Act (WMO), and it is compliant to ICH-GCP.

The NKI-AVL is the coordinating center. Part 1 is a mono-center study; for Part 2 extra centers are recruited (inter)nationally. The NKI-AVL Trial Office is the data center for the entire study. The NKI-AVL pharmacy is responsible for drug distribution and monitoring.

Patients are registered and allocated to a dose level or randomized to a treatment arm by the NKI-AVL trial office after signed informed consent is obtained. Patient data will be collected as described in the follow-up tables on case report forms according to the data management plan and checked according to the data monitoring and data validation plan. A statistical analysis plan will be used during data analysis. Toxicity data will be collected and graded according to CTCAE criteria and will be reported following national law. Response data will be collected and scored according to RECIST criteria. Patients will remain on treatment until progression or unacceptable toxicity. Procedures for dropout due to other reasons and patient replacement are in place.

## Discussion

Currently, *BRCA1* or *BRCA2* mutation carriers with breast cancer do not receive specific treatment targeting the defect in their tumor, despite strong preclinical and promising clinical evidence.

This study aims to provide evidence for a clinically meaningful improvement of the experimental regimen over standard of care chemotherapy for metastatic *BRCA1*- or *BRCA2*-mutated breast cancer. In choosing the experimental and control regimens, several choices had to be made. Carboplatin and olaparib share hematological toxicity, which is the main limiting factor in combination treatment. Preliminary evidence suggests that this combination cannot be administered for more than two cycles without treatment delays or unacceptable toxicity. The expected dose level contains efficacious doses of olaparib and carboplatin in a therapeutic range. Combining two modes of tumor cell killing may induce synergy requiring lower doses than single agent therapy [27]. The control arm treatments are all registered chemotherapy drugs for the treatment of advanced breast cancer and consist of capecitabine (after failure of anthracyclines and taxanes), paclitaxel (after failure of anthracyclines), eribulin (after failure of anthracyclines and taxanes), and vinorelbine (after failure of anthracyclines and taxanes) [34, 35, 37]. Capecitabine is a reasonable first choice for the expected population, which will consist of both ER-negative (*BRCA1* mutation enriched) and ER-positive (*BRCA2* mutation enriched) tumors, and has as an advantage that it is taken orally. We chose to enforce a homogeneous control group with one fixed comparator over physician’s best choice as first line treatment. Following recommendations of the “EMA Guideline on the evaluation of anticancer medicinal products in man,” we follow up patients in second line therapy to have an early indication if first line treatment has unfavorable effects on long-term survival [46]. Physician’s best choice in second line allows for tailoring the optimal drug out of the registered options, based on patient characteristics and pretreatment. At the moment no registered control regimen specifically targets a *BRCA1* or *BRCA2* defect. This precludes comparing carboplatin-olaparib to carboplatin alone if we only use registered choices. Crossover designs would ultimately only provide evidence on the sequence of applying carboplatin-olaparib or capecitabine in *BRCA1*- or *BRCA2*-mutated breast cancers but not on whether the regimen targeting the *BRCA* defect is better than a control treatment. In such a design it is also impossible to determine whether a DNA damage-inducing regimen in first line treatment may limit the efficacy of later lines of therapy.

The chosen design aims to provide evidence for a clinically meaningful improvement of carboplatin-olaparib followed by olaparib monotherapy over current standard therapy as first line treatment for *BRCA1*- or *BRCA2*-mutated advanced breast cancer. If this improvement is observed, the results can serve as a basis for registration of carboplatin-olaparib followed by olaparib monotherapy in combination with *BRCA1* or *BRCA2* mutation as biomarker.

### Trial status

We are currently recruiting patients for Part 1.

## Abbreviations

ALAT, alanine transaminase; ANC, absolute neutrophil count; ASAT, aspartate transaminase; AUC, area under the (time versus concentration) curve; BID, bis in die; *BRCA1*, breast cancer early onset 1; *BRCA2*, breast cancer early onset 2; CBG, College ter Beoordeling van Geneesmiddelen; CCMO, central committee on research involving human subjects; Cl, clearance; C_max_, maximum plasma concentration; CR, complete response; CRF, case report form; CTCAE, common terminology criteria for adverse events; CYP450, cytochrome P450; DLT, dose-limiting toxicity; DNA, deoxyribonucleic acid; DPD, dihydropyrimidine dehydrogenase; DSB, double strand break; ECG, electrocardiography; eCRF, electronic case report form; GCP, good clinical practice; GCSF, granulocyte colony-stimulating factor; Gy, gray; H2, histamine H2 receptor; HER2, human epidermal growth factor receptor 2; HIV, human immunodeficiency virus; ICH, International Conference on Harmonization; IV, intravenous; MEC, medical ethical committee; MRT, mean residence time; MTD, maximum tolerable dose; NCI, National Cancer Institute; NKI-AVL, Netherlands Cancer Institute - Antoni van Leeuwenhoek Hospital; ORR, objective response rate; OS, overall survival; PAR, poly(ADP)ribose; PARP1, poly(ADP)ribose polymerase 1; PBMC, peripheral mononuclear blood cells; PD, pharmacodynamics; PFS1, progression-free survival on first line treatment; PFS2, progression-free survival on second line treatment (measured from randomization); PK, pharmacokinetics; PR, partial response; PTC, Protocol Toetsingscommissie/Protocol Review Committee; q, quaque; RECIST, Response Evaluation Criteria In Solid Tumors; SD, stable disease; t_1/2_, half-life; t_max_, time to maximum plasma concentration; ULN, upper limit of normal; Vdd, volume of distribution; WHO, World Health Organization; wk, week; WMO, Wet Medisch-Wetenschappelijk Onderzoek met Mensen/Medical Research Involving Human Subjects Act

## Additional file

Additional file 1: Revival study SPIRIT checklist. Description of the trial protocol with the SPIRIT checklist. (DOC 122 kb)
